# Estimation of the rice aboveground biomass based on the first derivative spectrum and Boruta algorithm

**DOI:** 10.3389/fpls.2024.1396183

**Published:** 2024-04-25

**Authors:** Ying Nian, Xiangxiang Su, Hu Yue, Yongji Zhu, Jun Li, Weiqiang Wang, Yali Sheng, Qiang Ma, Jikai Liu, Xinwei Li

**Affiliations:** ^1^ College of Resource and Environment, Anhui Science and Technology University, Chuzhou, China; ^2^ Anhui Province Crop Intelligent Planting and Processing Technology Engineering Research Center, Anhui Science and Technology University, Chuzhou, Anhui, China; ^3^ Anhui Province Agricultural Waste Fertilizer Utilization and Cultivated Land Quality Improvement Engineering Research Center, Anhui Science and Technology University, Chuzhou, China

**Keywords:** rice, AGB, remote sensing, hyperspectral data, derivative transform, machine learning algorithm

## Abstract

Aboveground biomass (AGB) is regarded as a critical variable in monitoring crop growth and yield. The use of hyperspectral remote sensing has emerged as a viable method for the rapid and precise monitoring of AGB. Due to the extensive dimensionality and volume of hyperspectral data, it is crucial to effectively reduce data dimensionality and select sensitive spectral features to enhance the accuracy of rice AGB estimation models. At present, derivative transform and feature selection algorithms have become important means to solve this problem. However, few studies have systematically evaluated the impact of derivative spectrum combined with feature selection algorithm on rice AGB estimation. To this end, at the Xiaogang Village (Chuzhou City, China) Experimental Base in 2020, this study used an ASD FieldSpec handheld 2 ground spectrometer (Analytical Spectroscopy Devices, Boulder, Colorado, USA) to obtain canopy spectral data at the critical growth stage (tillering, jointing, booting, heading, and maturity stages) of rice, and evaluated the performance of the recursive feature elimination (RFE) and Boruta feature selection algorithm through partial least squares regression (PLSR), principal component regression (PCR), support vector machine (SVM) and ridge regression (RR). Moreover, we analyzed the importance of the optimal derivative spectrum. The findings indicate that (1) as the growth stage progresses, the correlation between rice canopy spectrum and AGB shows a trend from high to low, among which the first derivative spectrum (FD) has the strongest correlation with AGB. (2) The number of feature bands selected by the Boruta algorithm is 19~35, which has a good dimensionality reduction effect. (3) The combination of FD-Boruta-PCR (FB-PCR) demonstrated the best performance in estimating rice AGB, with an increase in R² of approximately 10% ~ 20% and a decrease in RMSE of approximately 0.08% ~ 14%. (4) The best estimation stage is the booting stage, with R^2^ values between 0.60 and 0.74 and RMSE values between 1288.23 and 1554.82 kg/hm^2^. This study confirms the accuracy of hyperspectral remote sensing in estimating vegetation biomass and further explores the theoretical foundation and future direction for monitoring rice growth dynamics.

## 1 Introduction

Rice (*Oryza sativa*), a herbaceous plant belonging to the genus *Oryza*, has a long history of cultivation and a broad planting region (Krishnan et al., 2011). Aboveground biomass (AGB) serves as a critical indicator for describing crop growth (Wang et al., 2016). Applying scientific methods to estimate rice AGB to improve crop yields is crucial for human food security and increased economic benefits (Spiertz and Ewert, 2009; Devia et al., 2019; Cao et al., 2021b). The conventional method for acquiring AGB primarily involves field sampling, which is both time-consuming and laborious (Gnyp et al., 2014). In contrast, remote sensing technology provides a fast and nondestructive method, providing a scientific basis and technical support for accurate estimation of AGB (Schino et al., 2003). Among them, satellite remote sensing platforms is commonly used for AGB estimation in large areas, such as forests (Lucas et al., 2020), grasslands (Fang et al., 2018; Yang et al., 2018), However, it is easily affected by spatial conditions and it is difficult to obtain satisfactory results at small field scales (Yue et al., 2019). UAV remote sensing platforms can perform low-altitude operations and obtain high-resolution images of crops, but the spectral resolution is low, which is not conducive to in-depth exploration of the spectral response of crops (Bansod et al., 2017). In comparison, near-ground hyperspectral remote sensing is not only suitable for small-scale crop monitoring, but can also acquire fine spectral data with high resolution and a broad spectrum of wavelengths, encapsulating abundant crop phenotype information and thereby offering increased opportunities for estimating the physical and chemical parameters of vegetation (Galvão et al., 2005; Atzberger et al., 2015).

To enhance the AGB estimation performance via hyperspectral remote sensing, the original spectral data are typically preprocessed to reduce noise and increase the accuracy of the data (Mishra et al., 2020). The Savitzky–Golay (SG) smoothing algorithm and derivative transform are the principal methods for preprocessing. The SG smoothing algorithm helps to mitigate interference signals within spectral data and minimize random errors, thereby improving data reliability (Savitzky and Golay, 1964; Liu et al., 2014). Derivative transform spectrum emphasizes the absorption and reflection characteristics of the subject matter and augments the differentiation in spectral information (Gerretzen et al., 2015). For instance, Wang et al. (2004) demonstrated that the derivative spectrum can lessen the impact of noise, exhibits a high correlation with rice AGB, and performs effectively in estimating rice AGB. However, spectral data still face challenges such as excessive information redundancy and high dimensionality after preprocessing. There is a need for a reliable method that can reduce the dimensionality of spectral data and eliminate high correlations between bands.

In previous research, Some researchers have used a single vegetation index to estimate rice AGB and achieved good results. However, the vegetation indices is usually a combination of several sensitive wavelengths, ignoring a large volume of spectral information. In the 2020s, some scholars adopted complex dimensionality reduction methods to improve the utilization efficiency of rice spectral data. For example, Jia et al. (2022) used the continuous projection algorithm (SPA) to reduce the hyperspectral dimension and identified 12 characteristic bands to estimate the soil plant analysis development (SPAD) content of rice. Yu et al. (2020) utilized principal component analysis (PCA) to extract five principal components for the inversion of rice leaf nitrogen deficiency. The above studies used different dimensionality reduction methods to reduce spectral data redundancy, which is of positive significance for applying hyperspectral remote sensing technology to estimate rice AGB accurately. However, they present limitations. The objective of SPA is to eliminate combinations of variables that exhibit low collinearity and include distinctive information; however, this approach might omit some initial characteristics in the spectral data (Cao et al., 2021a). PCA is capable of using several independent principal components to depict the original data, yet this technique is confined to linear projection and exhibits inadequate performance in managing nonlinear data relationships (Moore, 1981; Abdi and Williams, 2010). Consequently, the effectiveness of the monitoring model in practical scenarios may be affected by different dimensionality reduction methods (Fu et al., 2020). Given these considerations, selecting a suitable dimensionality reduction method is essential for accurately identifying sensitive bands and enhancing the accuracy of the estimation model.

As a data dimensionality reduction method, the core of Boruta algorithm is to find all feature bands related to the dependent variable. Compared with other commonly used feature screening algorithms, Compared with other commonly used feature screening algorithms (Kursa and Rudnicki, 2010; Kursa et al., 2010). The algorithm was originally used in biological and medical fields and has since been used in agricultural research as well. For instance, Li et al. (2022) demonstrated that filtering spectral indices from winter wheat canopy hyperspectral data using the Boruta algorithm enhances data validity and establishes an accurate yield estimation model. The Boruta algorithm has great potential in physical and chemical parameter estimation. To further evaluate the ability of the Boruta algorithm to estimate rice AGB, this study also selected the RFE feature selection algorithm for comparison. RFE, an adaptive feature selection algorithm, iteratively removes the least important feature variables and ranks feature importance until the optimal feature subset is identified. This approach minimizes the effects of random fluctuations and interference information (Tunca et al., 2023). Yoosefzadeh-Najafabadi et al. (2021) applied RFE to determine the optimal wavelengths from hyperspectral data for soybean yield prediction. This indicates that these feature selection methods are effective in improving estimation models’ accuracy. However, relatively few studies have utilized the Boruta algorithm and the RFE algorithm for feature selection of derivative spectra to construct rice AGB estimation models.

Therefore, this study used the Boruta and RFE algorithms to eliminate interference information, identify sensitive bands, and integrate partial least squares regression (PLSR), principal component regression (PCR), support vector machine (SVM) and ridge regression (RR) to develop an AGB estimation model. Additionally, this study compares the estimation effects of various combinations of dimensionality reduction algorithms and machine learning models to identify the most accurate estimation results. The research objectives were to (1) compare the correlations between derivative spectrum of different orders and rice AGB to determine the optimal order; (2) evaluate the effectiveness of the Boruta algorithm and RFE algorithm in selecting feature bands to determine the best dimensionality reduction method; (3) evaluate the estimation capability of machine learning algorithms combined with feature selection algorithms on rice AGB; and (4) identify the optimal combination of models and the most suitable estimation stage.

## 2 Materials and methods

### 2.1 Experimental design

**Figure 1:**
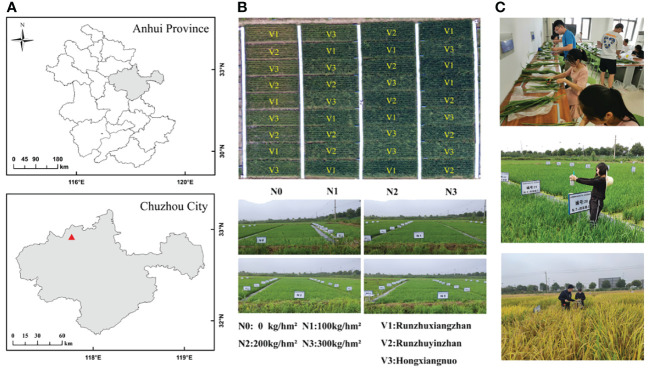
Rice experimental area **(A, B)** and data collection **(C)**.

The study was conducted in Xiaogang village, Fengyang County, Chuzhou city, Anhui Province, China (longitude 117°46′7″E, latitude 32°48′52″N) (**Figure 1A**), which is characterized by a subtropical monsoon climate, an average annual temperature of 15.4°C, and an average annual precipitation of 1179.2 mm. The soil predominantly consists of clay with medium fertility on flat terrain. The experimental area included 36 plots, measuring 2 m × 8 m each. The rice varieties tested were Runzhuxiangzhan (V1), Runzhuyinzhan (V2), and Hongxiangnuo (V3), with each plot replicated three times. Four nitrogen gradient treatments were applied with nitrogen application rates of N0 (0 kg/hm²), N1 (100 kg/hm²), N2 (200 kg/hm²), and N3 (300 kg/hm²). Phosphorus (90 kg/hm²) and potash (135 kg/hm²) fertilizers were applied once as basal fertilizer (**Figure 1B**). The experiment spanned from June 2020 to October 2020. The plants experienced no drought or flooding during the seedling stage and favorable conditions of abundant sunshine and moderate temperatures during the mid-growth stage, which are conducive to rice growth and development. Field management practices followed local cultivation methods and pest control technologies.

### 2.2 Data acquisition

#### 2.2.1 Ground data acquisition

Rice samples were collected on July 25 (tillering stage), August 13 (jointing stage), August 23 (booting stage), August 31 (heading stage) and September 20 (maturity stage). The sampling time was consistent with the hyperspectral data acquisition time. Rice samples were collected from three holes in each plot, and the locations of the sampling points were marked after each sampling to avoid duplicate sampling.

Rice samples were subsequently sent to the laboratory, after which stem, leaf, and ear separation was performed (**Figure 1C**). The sample was placed in an oven, dried for 0.5 h after the temperature increased to 105°C, and then adjusted to 75°C for more than 24 h until the mass was constant. The dry mass of the sample was obtained by summing the dry mass of the stem, leaves and ears of the plant. Finally, the aboveground biomass of the rice plants in each plot was obtained through the dry mass of the sample and the row spacing during rice planting.

#### 2.2.2 Hyperspectral data collection

Hyperspectral data on the rice canopy spectra were collected using a handheld ground spectrometer (ASD FieldSpec® HandHeld™2) covering a wavelength range from 325 to 1075 nm with a resolution of 1 nm. Measurements were conducted between 10:00 a.m. and 2:00 p.m. for each test. Prior to each measurement session, the instrument was calibrated against a standard radiation white plate and recalibrated after every two blocks to ensure accuracy. The data were collected at three evenly distributed points along the diagonal of each plot, with the spectrometer positioned vertically 0.5 m above the rice canopy (**Figure 1C**). The spectral data from the same plot were processed collectively, and the average spectral reflectance of the plot was calculated.

### 2.3 Hyperspectral data processing

#### 2.3.1 SG smoothing and derivative transform

The Savitzky−Golay (SG) smoothing algorithm, a digital signal processing technique proposed by Savitzky and Golay, is employed to enhance signal frequency and eliminate data noise (Savitzky and Golay, 1964). The smoothing effect of the algorithm, which varies with the window length, preserves the original shape and peak heights of the wave signal (Schafer, 2011). To minimize data fluctuations, capture the overall trend of the spectral curves, and accurately analyze the spectral characteristics of rice, this study applied SG smoothing to the canopy spectral curves from 325 to 1075 nm recorded during the growth stage (Chen et al., 2020; Shi et al., 2021). However, due to the influence of noise, only the 400 to 900 nm range was selected for analysis.

Derivative transform serves to diminish background signals, with the FD highlighting absorption peaks and shoulders in the original spectrum (OS) (Demetriades-Shah et al., 1990; Becker et al., 2005). By differentiating peaks and valleys, more precise spectral reflectance data can be obtained (Ji et al., 2022; Zhao et al., 2022). The second derivative spectrum (SD) facilitates the accurate identification of absorption peak and shoulder positions within the OS, enhancing spectral reflectance differentiation and eliminating baseline offsets in spectral reflectance data (Yang et al., 2011). This study utilized the derivative function within the Origin software’s data processing menu (Origin 2018) for these transformations.

#### 2.3.2 Feature selection

Feature selection algorithms play a pivotal role in optimizing datasets prior to model construction. In this investigation, both the Boruta algorithm and the RFE algorithm were employed for feature selection.

The Boruta algorithm (Prasad et al., 2019) is an innovative feature selection method derived from the random forest approach. It identifies all characteristic bands in spectral data that are related to dependent variables, thereby elucidating the relationship between spectral characteristics and rice AGB. The foundational concept involves shuffling the original parameters to create shadow parameters, which are then combined with the original parameters into a feature matrix for training. Based on the importance scores from the random forest, the shadow parameters are ranked by importance, with the highest value designated the Z score. Original parameters more significant than the Z score are labeled “most important”, while those below are deemed “unimportant” and thus eliminated. Ultimately, the most important original parameters are selected as the optimal combination for constructing the inversion model.

Recursive feature elimination (RFE) (Guyon et al., 2002) is a wrapper-based selection method that starts with the construction of an initial model, ranking all feature bands by their importance, and iteratively removing the least significant features. By retraining the model with the remaining features and repeating this process, it gradually identifies the most critical subset of features. This algorithm excels by iteratively evaluating the contribution of each feature band to the model, thereby identifying the optimal feature subset to reveal the underlying structure and patterns within the data.

### 2.4 Model construction method

To thoroughly investigate the relationships between the independent and dependent variables, four machine learning models were selected for comparison: PLSR, PCR, SVM, and RR.

PLSR (Geladi and Kowalski, 1986) is a multivariate linear statistical method that focuses on finding a hyperplane that minimizes the variance between the response and independent variables. It projects predictor and observed variables into a new space to derive a linear regression model, known as a bilinear factor model, due to the presence of both data X and Y in this new space.

PCR (Kawano et al., 2018) employs PCA for predictive data mining, transforming original variables into principal components that are linear combinations of the original variables and mutually independent. Regression analysis is then performed on these principal components to derive the regression equation, which is subsequently applied to the original variables.

SVM (Suykens and Vandewalle, 1999) operates as a supervised learning model utilized for analyzing data in classification and regression analysis. The core concept involves identifying the optimal classification hyperplane for two types of samples in the original space when they are linearly separable. In instances where linear separability is not achievable, the samples are projected into a high-dimensional feature space where an optimal hyperplane is determined. This hyperplane classifies the samples, aiming to minimize the distance within similar classes and maximize the distance between distinct classes.

RR (Ivanda et al., 2021) serves as a technique for estimating the coefficients of a multiple regression model in scenarios where the independent variables exhibit high correlation. This is achieved by incorporating an L2 regularization term into the OLS loss function. The L2 regularization term is calculated as the product of the sum of the squares of the coefficients and a regularization parameter λ (lambda).

### 2.5 Accuracy evaluation

In this study, 70% of the sample data (n=25) were selected as the modeling set for each growth stage, and 30% of the sample data (n=11) were used as the validation set to construct the rice AGB estimation model. The coefficient of determination (R²), root mean square error (RMSE), and mean absolute error (MAE) were used to evaluate the model accuracy. The closer R² is to 1, the lower the RMSE and MAE are, and the higher the accuracy of the estimation model is. R², RMSE and MAE are calculated using Equations 1–3, respectively:


(1) $${\text{R}}^{2}=1-\frac{\sum_{\text{i}=1}^{\text{n}}{\left({\text{x}}_{\text{i}}-{\text{y}}_{\text{i}}\right)}^{2}}{\sum_{\text{i}=1}^{\text{n}}{\left({\text{x}}_{\text{i}}-\hat{x}\right)}^{2}}$$

(2) $$\text{RMSE}=\sqrt{\frac{\sum_{\text{i}=1}^{\text{n}}{\left({\text{y}}_{\text{i}}-{\text{x}}_{\text{i}}\right)}^{2}}{\text{n}}\;}$$

(3) $$\text{MAE}=\frac{\sum_{\text{i}=1}^{\text{n}}\left|{\text{y}}_{\text{i}}-{\text{x}}_{\text{i}}\right|}{\text{n}}$$

Note: ${\text{x}}_{\text{i}}$ , ${\text{y}}_{\text{i}}$ , and $\hat{x}$ are the actual measured value, predicted value and average value of the measured data, respectively; n is the number of samples.

## 3 Results

**Figure 2:**
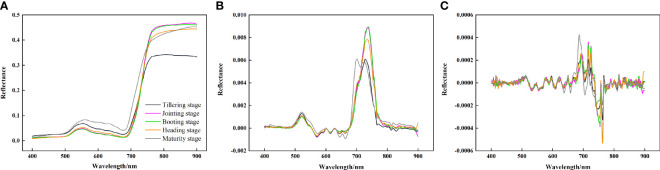
Derivative transformation spectral curves of rice canopy at different growth stages. **(A)** original spectrum, **(B)** first derivative spectrum, **(C)** second derivative spectrum.

SG smoothing was applied to the spectral curves within the 400 to 900 nm range, The processing results of OS, FD and SD at different growth stages are shown in **Figure 2**.

The processing results for the OS reveal the characteristic spectral profile of the rice canopy, marked by typical green plant reflectance features, with spectral reflectance values ranging from 0 to 0.5. Notably, a reflection peak and an absorption valley are observed near wavelengths of 550 nm and 680 nm, respectively, both of which are situated within the visible light spectrum. The spectral reflectance of rice rapidly increases within the 680 to 750 nm range, creating a “red edge” phenomenon (Gitelson et al., 1996). The first peak of the FD curve appears at 500 ~ 550 nm. The band range of 680 ~ 750 nm shows a drastic change that first rises and then falls. The SD curve exhibits continuous fluctuations across the 500 to 690 nm band range, with two notable peaks within the 700 to 800 nm band range.

### 3.1 Correlations between different orders of derivative spectrum and rice AGB

**Figure 3:**
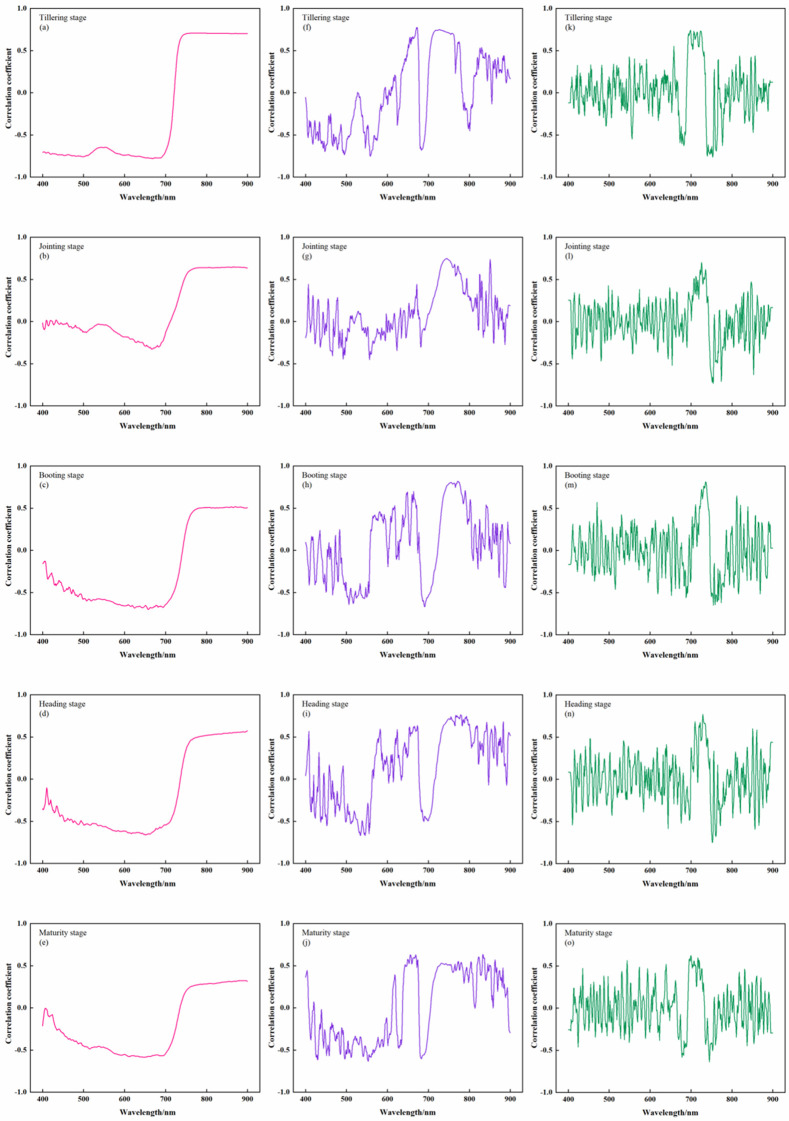
Correlation between rice canopy spectra and AGB at different growth stages. **(A-E)** The correlation between OS and AGB; **(F-J)** the correlation between FD and AGB; **(K-O)** the correlation between SD and AGB.

To fully explore the sensitivity of the different orders of rice canopy spectra and to compare the effects of spectral transformations on AGB, FD and SD transformations were carried out on the basis of OS, and their correlations with rice AGB were investigated (**Figure 3**).

During the full growth stage, the correlation between OS and AGB showed a consistent trend from negative to positive as the band increased, with the maximum correlation at 720 nm. The correlation curve between FD and AGB shows a continuous fluctuation trend in the 400 ~ 900 nm band, among which the 680 ~ 750 nm band has the highest correlation, with a correlation coefficient between 0.60 ~ 0.85. The correlation curve between SD and AGB was similar to that between FD and AGB, but the fluctuations were more severe.

**Figure 4:**
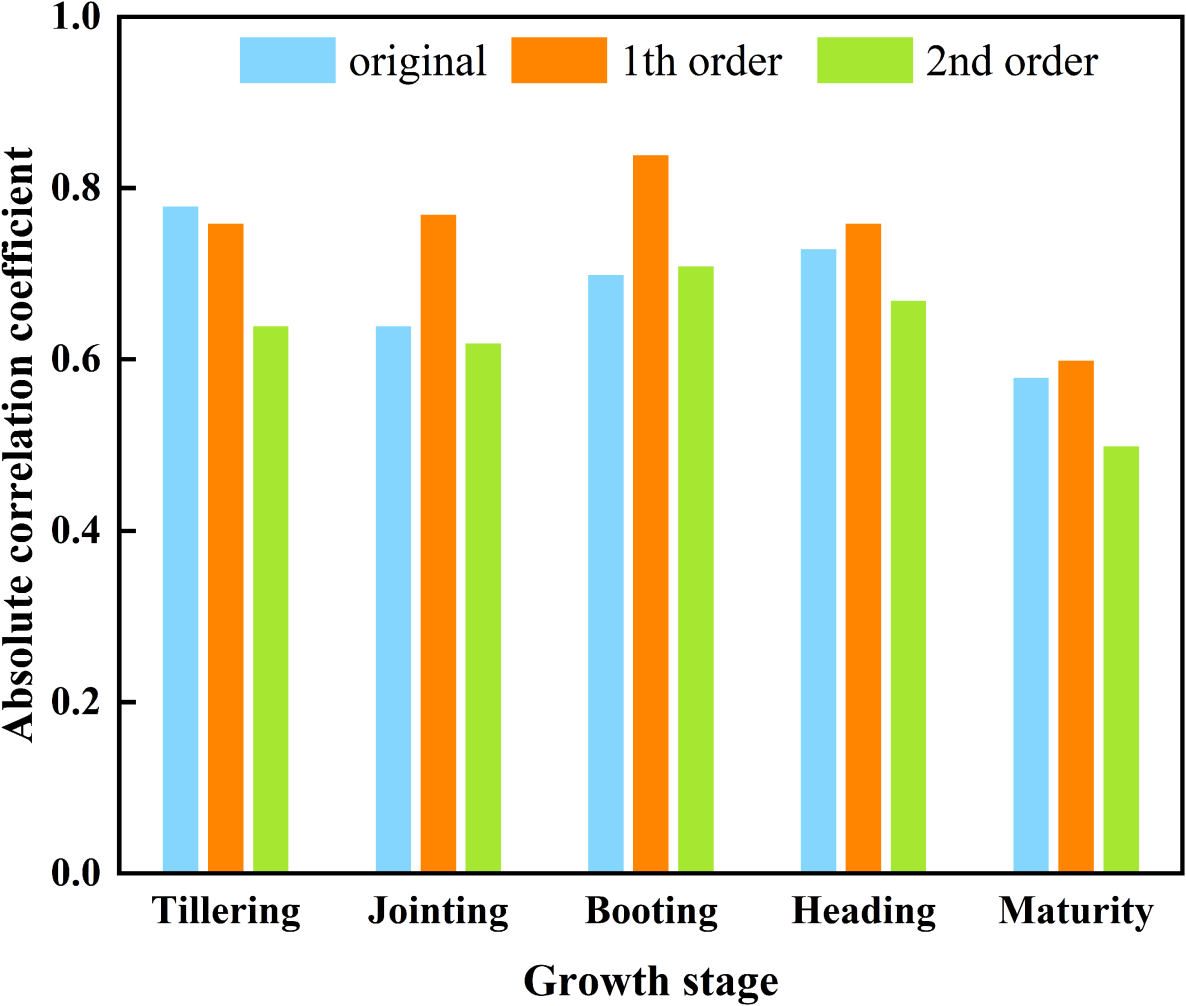
The maximum absolute value of the correlation coefficient between different order derivative spectra and AGB.

In summary, the derivative spectrum of the different orders were compared with the maximum absolute value of the AGB correlation coefficient (**Figure 4**). Except for the tillering stage, the maximum values in the other four periods all occurred at FD. Comprehensive analysis revealed that the FD could better reflect the growth status of rice, so the FD was used as the input variable of the feature screening algorithm for subsequent construction of the AGB estimation model.

### 3.2 Feature band selection

An AGB estimation model utilizing PLSR, PCR, SVM, and RR was developed to evaluate the effectiveness of the band screening algorithms. This model incorporated characteristic bands selected by both the Boruta algorithm and the RFE algorithm, as well as the full band as input variables. The diversity and scope of the characteristic bands selected by these feature screening algorithms during different growth stages are summarized in **Table 1**. Detailed feature selection results are shown in **Appendix 2**.

**Table 1:** Comparison of feature selection results between the Boruta algorithm and RFE algorithm.

Feature selection algorithm	Growth Stage	Number	Feature band range (nm)
Boruta	Tillering	19	724 ~ 761
	Jointing	35	644, 721 ~ 771, 816, 817
	Booting	27	664 ~ 694, 717 ~ 774
	Heading	24	414, 416, 699 ~ 777,817, 873
	Maturity	30	420, 498, 686 ~ 759, 842
RFE	Tillering	3	742, 751, 761
	Jointing	392	402 ~ 900
	Booting	3	753, 754, 764
	Heading	127	402 ~ 495, 507 ~ 594, 614 ~ 699, 702 ~ 900
	Maturity	439	400 ~ 900

The Boruta algorithm demonstrated remarkable stability, with the number of characteristic wavelengths in each growth stage not exceeding 40. In contrast, the RFE algorithm showed more significant fluctuations, with 3 characteristic wavelengths identified at both the tillering and booting stages and 439 at the maturity stage, indicating substantial variance in the number of wavelengths identified across different growth stages. Notably, the characteristic wavelengths identified by both screening algorithms predominantly fall within the “red edge” spectral range, highlighting their importance in estimating rice AGB.

### 3.3 Establishment and evaluation of the rice AGB estimation model based on FD

**Figure 5:**
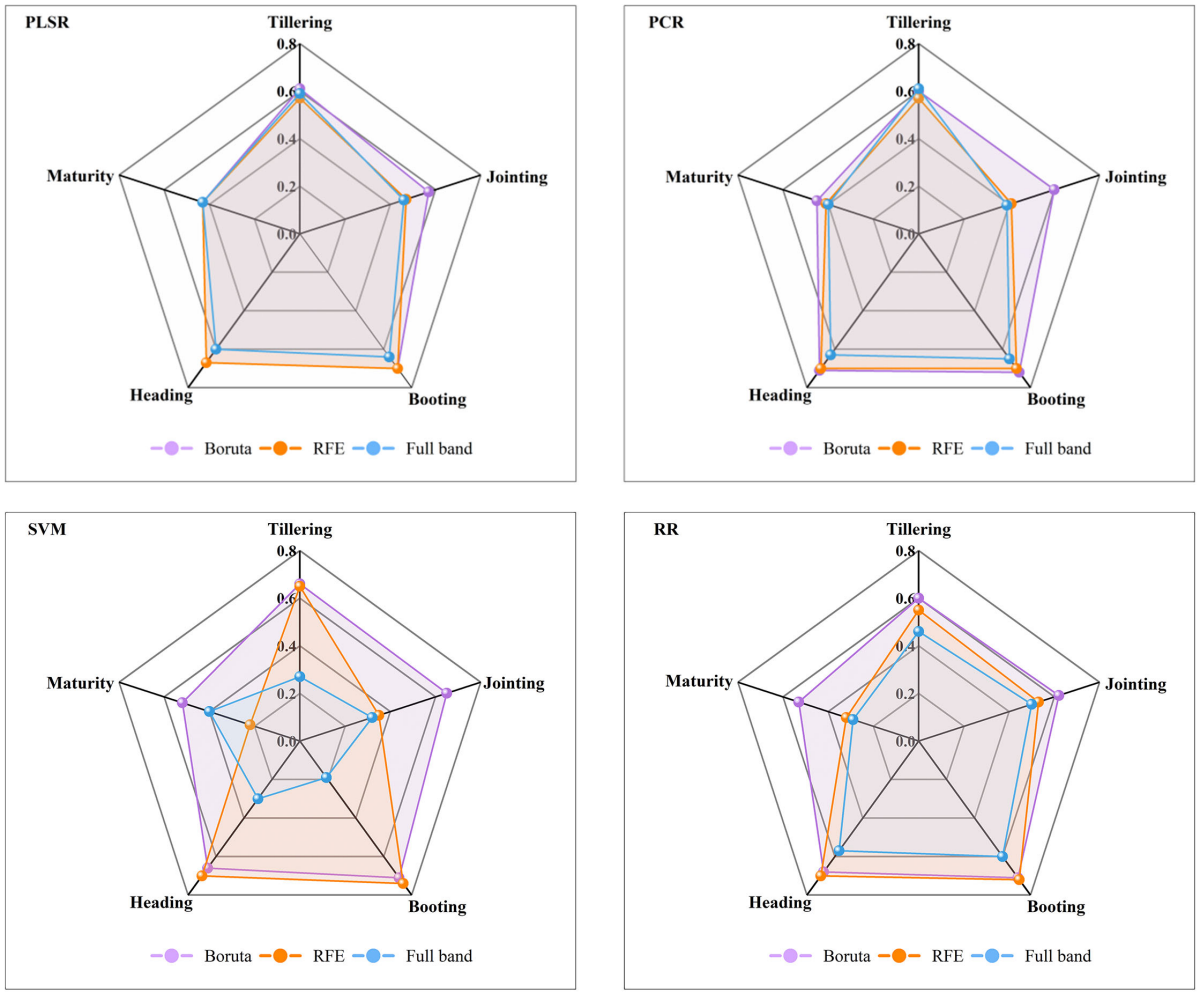
Comparison of the R² values of the different estimation models for the validation sets. PLSR, partial least squares regression; PCR, principal component regression; SVM, support vector machine; RR, ridge regression.

This study employs three types of model input variables: first derivative full band (FD-FB), the characteristic bands selected by the Boruta algorithm (FD-Boruta), and the characteristic bands selected by the RFE algorithm (FD-RFE). Sixty AGB estimation models were developed for various growth stages using four machine learning algorithms (PLSR, PCR, SVM, RR), with R² (**Figure 5**), RMSE (**Table 2**) and MAE (**Appendix 1**) serving as the model evaluation metrics.

**Table 2:** Rice AGB estimation model under different machine learning algorithms (R^2^, RMSE).

machine learning algorithm	Bandselection algorithm	Tillering				Jointing				Booting				Heading				Maturity			
		Modeling Set		Validation Set		Modeling Set		Validation Set		Modeling Set		Validation Set		Modeling Set		ValidationSet		Modeling Set		Validation Set	
		R²	RMSE(kg/hm²)	R²	RMSE(kg/hm²)	R²	RMSE(kg/hm²)	R²	RMSE(kg/hm²)	R²	RMSE(kg/hm²)	R²	RMSE(kg/hm²)	R²	RMSE(kg/hm²)	R²	RMSE(kg/hm²)	R²	RMSE(kg/hm²)	R²	RMSE(kg/hm²)
PLSR	Boruta	0.54	427.85	0.61	464.34	0.68	801.67	0.57	1072.49	0.72	1096.66	0.70	1351.62	0.72	1125.80	0.67	1405.25	0.49	2017.00	0.43	2515.77
	RFE	0.53	434.66	0.57	493.81	0.78	635.91	0.47	1226.82	0.70	1148.63	0.70	1336.75	0.80	908.01	0.67	1398.94	0.63	1736.85	0.43	2407.61
	FB	0.72	324.48	0.59	473.29	0.77	644.07	0.46	1245.08	0.81	912.34	0.64	1474.44	0.79	948.73	0.60	1547.85	0.63	1744.52	0.43	2406.05
PCR	Boruta	0.54	427.33	0.60	469.15	0.64	853.54	0.60	1057.88	0.72	1101.43	0.72	1290.24	0.70	1171.49	0.71	1307.01	0.45	2120.01	0.45	2392.65
	RFE	0.53	435.68	0.57	493.77	0.43	1067.73	0.41	1311.00	0.70	1150.33	0.70	1338.99	0.70	1180.64	0.70	1321.14	0.43	2178.91	0.41	2412.86
	FB	0.55	423.94	0.61	477.47	0.41	1087.71	0.39	1332.82	0.68	1186.97	0.65	1465.01	0.59	1377.74	0.63	1541.66	0.41	2205.87	0.40	2430.66
SVM	Boruta	0.67	368.29	0.66	434.33	0.80	663.56	0.65	985.55	0.86	800.08	0.71	1349.87	0.82	902.99	0.66	1411.43	0.85	1135.73	0.52	2189.95
	RFE	0.67	367.30	0.65	445.05	0.97	329.32	0.35	1370.60	0.75	1073.13	0.74	1311.04	0.99	216.76	0.70	1430.80	0.96	716.15	0.22	2964.74
	FB	0.80	481.27	0.27	701.52	0.98	276.86	0.32	1412.37	0.97	486.02	0.19	2316.48	0.98	386.45	0.30	2266.78	0.95	852.86	0.19	2980.00
RR	Boruta	0.58	421.84	0.60	504.74	0.72	764.35	0.62	1009.78	0.76	1036.43	0.71	1301.00	0.81	955.61	0.68	1370.52	0.63	1827.13	0.53	2217.62
	RFE	0.53	435.50	0.55	513.04	0.90	513.16	0.53	1120.75	0.70	1144.30	0.72	1288.23	0.94	579.73	0.70	1318.70	0.91	1102.65	0.32	2628.54
	FB	0.95	192.63	0.46	514.86	0.90	518.87	0.50	1160.32	0.94	627.07	0.60	1554.82	0.95	612.77	0.57	1588.79	0.92	1095.07	0.29	2648.03

PLSR, partial least squares regression; PCR, principal component regression; SVM, support vector machine; RR, ridge regression.

Initially, the AGB estimation abilities of different feature selection algorithms were compared within the same model framework. In the PLSR model, the estimation precision for FD-Boruta and FD-RFE was found to be comparable, with R² values ranging from 0.43 to 0.70, and RMSE values between 464.34 and 2515.77 kg/hm² and 493.81 and 2407.61 kg/hm², respectively. These results are more accurate than those obtained using FD-FB, with R² values improving by 0 to 0.06 and RMSE values decreasing by 29.47 to 148.91 kg/hm². Among the PCR models, FD-Boruta exhibited the highest estimation accuracy, followed by FD-RFE, with FD-FB showing the least accuracy. The R² accuracy of FD-Boruta improved by 0.03, 0.19, 0.02, 0.01, and 0.04, respectively, compared to FD-RFE. In contrast, the R² accuracy of FD-RFE improved by 0.02, 0.05, 0.07, and 0.01 compared to FD-FB. The outcomes for the SVM and RR models were analogous, although the performances of the three algorithms varied significantly. R² ranged from 0.58 to 0.71 for FD-Boruta, 0.22 to 0.74 for FD-RFE, and 0.19 to 0.60 for FD-FB.

Second, the estimation accuracy of AGB by different models under the same feature selection algorithm was compared. For the model constructed with FD-Boruta inputs, when the estimation accuracy was similar, FD-Boruta-PCR demonstrated more stable performance. The estimation outcomes of models based on FD-RFE varied significantly. FD-RFE-PLSR and FD-RFE-PCR provided more precise estimates of rice AGB, whereas the FD-RFE-SVM and FD-RFE-RR models exhibited overfitting in certain stages. The models based on FD-RFE generally showed poor performance, with model R² values below 0.65, and the accuracy of the FD-FB-SVM and FD-FB-RR models varied greatly.

Finally, the performance of all model combinations was assessed for estimating AGB effects across different growth stages. In the tillering stage, FD-Boruta-SVM yielded the best estimation, with an R² of 0.66 and an RMSE of 34.33 kg/hm². During the jointing stage, FD-Boruta-SVM achieved the highest estimation accuracy, with an R² of 0.65 and an RMSE of 985.55 kg/hm², while FD-FB-SVM showed the lowest accuracy, with an R² of 0.32 and an RMSE of 1412.37 kg/hm2. In the booting stage, the estimation accuracies R² of models using FD-FB and FD-RFE inputs were both above 0.7, with FD-RFE-SVM and FD-Boruta-PCR performing the best, achieving R² values of 0.74 and 0.72, respectively, and RMSE values of 1311.04 kg/hm² and 1290.24 kg/hm², respectively. At the heading stage, FD-Boruta-PCR had the highest estimation accuracy, with an R² of 0.71, while FD-FB-SVM had the lowest accuracy, with an R² of 0.30. In the maturity stage, the AGB estimation accuracy of all the models decreased, with R² values ranging from 0.19 to 0.53.

In conclusion, models built using the Boruta algorithm are more reliable and exhibit greater stability, among which FD-Boruta-PCR is the most stable, followed by FD-Boruta-PLSR. Considering the model estimation accuracy, the booting period is the most accurately estimated, with R² values exceeding 0.7.

## 4 Discussion

### 4.1 Correlations between OS, FD, SD and AGB

An in-depth analysis of the correlation between spectral information and AGB will facilitate a comprehensive understanding of the growth status of rice. This study performed a correlation analysis between different orders of derivative spectrum and AGB at different growth stages of rice. Generally, the correlation between the derivative spectrum of various orders and AGB tended to increase and then decrease throughout the growth stage, potentially due to the influence of the rice growth cycle (Xu et al., 2023). From the tillering stage to heading stage, as the chlorophyll content in rice leaves increases and the vegetation canopy structure becomes fully developed, the canopy becomes richer in spectral information, which can more accurately reflect crop characteristics (Ren et al., 2018). In the mature stage, as stems and leaves progressively wither and yellow, the chlorophyll content drops sharply, the spectral characteristics obtained are less able to accurately represent crop growth, leading to a decrease in the correlation between the canopy spectrum and AGB (Yang and Chen, 2004; He et al., 2019; Zhang et al., 2019).

Within the 680~750 nm band range, the correlation between the canopy spectrum and AGB reached its maximum value in different stages (**Figure 3**). This occurs because this band serves as the transition region between the infrared and near-infrared bands, where spectral reflectance transitions rapidly from a low negative correlation to a high positive correlation. This shift is attributed to strong absorption and reflection (Kanke et al., 2016; Xu et al., 2022). The correlation between FD and AGB was generally greater than that between OS and SD at the different fertility stages (**Figure 4**). This observation aligns with the findings of Liang et al. (2018) and Tong et al. (2023). As a method for analyzing spectral information, derivative transform can diminish noise and enhance data accuracy (Li and Xie, 2015). FD reflects the slope of the spectrum, while SD represents the change in the slope of the reflection spectrum. While SD identifies more absorption peaks, it also introduces noise and may result in errors (Shen et al., 2022). Therefore, FD is strongly correlated with AGB and can be effectively utilized for estimating rice AGB.

### 4.2 Rice AGB estimation based on different feature selection algorithms

In the realm of feature selection, prior research has demonstrated that employing screened feature variables for model construction enhances the estimation power, inversion accuracy, and utility of the original models (Gao et al., 2019; Sun et al., 2021). For example, Yu et al. (2023) used the SPA algorithm to extract sensitive bands in rice canopy spectral data, and combined with PROSAIL to establish a rice AGB estimation model, achieving high accuracy (R²=0.69). This study yielded similar findings, the characteristic band had a greater estimation ability than the original band. The difference is that previous research is usually based on OS and does not involve derivative transform. Therefore, whether the estimation model constructed by derivative transformed spectrum is better than OS remains to be determined. This study performs SG smoothing and derivative transform on OS and selects characteristic bands based on the optimal derivative spectrum. The results show that the Boruta algorithm is more robust than the RFE algorithm, and the selected feature bands are more sensitive, which is conducive to constructing subsequent rice AGB estimation model.

Under identical conditions, the most accurate AGB estimates were achieved using feature bands selected by the Boruta algorithm. This superiority of the Boruta algorithm is attributed to its ability to identify bands of features that are genuinely relevant to the dependent variable, thereby enhancing the prediction accuracy of the model (Kursa and Rudnicki, 2010), a conclusion that aligns with the findings of Degenhardt et al. (2019). However, the RFE algorithm exhibited suboptimal performance in the jointing, heading, and maturity stages. This could be due to the RFE algorithm generates feature subsets with corresponding accuracy by continuously building models. This process may result in retaining a large number of features, leading to significant collinearity between bands and diminishing the model’s estimation accuracy (Paul et al., 2015; Chen et al., 2018), echoing the research results of Lin et al. (2023). The poorest performance was observed when the entire band was utilized as an input variable, attributed to the presence of redundant information and increased collinearity among bands, which impaired the model’s estimation capability (Hansen and Schjoerring, 2003; Pan et al., 2017).

Compared with the RFE algorithm, the Boruta algorithm identified fewer feature bands (**Table 1**); however, the resulting estimation model was more stable. This stability may be due to the characteristic bands determined by the Boruta algorithm being more effective and independent (Poona et al., 2016). Additionally, when comparing feature bands screened by both the Boruta and RFE algorithms, it was noted that their intersection predominantly occurred in the “red edge” region. This could be due to the band encapsulates rich crop information and is highly correlated with AGB (Horler et al., 1983; Filella and Penuelas, 1994), consistent with the findings of Cheng et al. (2017), who assessed the estimation effects of eight vegetation indices on rice canopy composition AGB and found the red edge index to be particularly sensitive to leaf AGB, achieving the highest estimation accuracy.

Another aspect of interest is that the characteristic bands identified by the Boruta algorithm, which are relatively evenly distributed across the spectrum, are predominantly found at wavelengths of 420 nm, 644 nm, 750 nm, and 842 nm. These characteristic bands are mainly located in areas sensitive to crop AGB response and are relatively evenly distributed. During the tillering stage, the characteristic bands are primarily located in the “red edge” region, which may be due to the low vegetation coverage at this time and the spectrum response is not obvious (Kokaly and Clark, 1999; Clevers et al., 2001). In the jointing stage, characteristic wavelengths are observed in the near-infrared region, likely due to the increase in rice canopy biomass, making the wavelength response in this region more pronounced and easier to detect during the selection process (Datt, 1998). From the heading to the maturity stage, a few characteristic wavelengths in the blue light region are identified, possibly because the chlorophyll content in the leaves gradually decreases, leading to increased spectral reflectance in the blue light region (Yan-lin et al., 2004). Thus, the Boruta algorithm effectively mines the spectral information of the rice canopy, and the selected characteristic bands align with the spectral response changes in the rice canopy, thereby enhancing the accuracy of AGB estimation.

### 4.3 Rice AGB estimation based on different machine learning algorithms

The performances of various models were meticulously compared, with the PCR model emerging as the most reliable and stable for estimating AGB. The differences between the R^2^ values for the modeling set and the validation set are minimal, ranging from 0 to 0.1. Both the RMSE and MAE are lower for the PCR model than for the other models. This superior performance of the PCR model can be attributed to its foundation in predictive data mining technology through principal component analysis (PCA), which efficiently reduces the dimensionality of independent variables, mitigates the effects of multicollinearity among variables, and enhances the model’s adaptability (Suryakala and Prince, 2019). In the PLSR model. Models constructed with three different input variables all demonstrated the ability to estimate rice AGB, particularly at the booting stage, when the R² of the validation set exceeded 0.6. This performance is likely due to the robust adaptability of the PLSR algorithm, its ability to diminish the dimensionality of spectral data, its ability to effectively handle complex collinear relationships between independent variables, and its overall enhancement of the model’s estimation accuracy (Helland, 2001). In the SVM model, estimation models built on FD-RFE and FD-FB inputs exhibited overfitting at the jointing, heading, and maturity stages. Due to the excessive number of FD-RFE and FD-FB, when fitting as sample data, the noise interference in the model is large, resulting in an increase in estimation error. Even some noise may be recognized as characteristic frequency bands by machine learning algorithms, destroying the preset classification rules and significantly affecting the accuracy of model estimation (Cherkassky, 1997; Shafaei and Kisi, 2017; Raja et al., 2022). FD-Boruta has a small number but high estimation potential and can solve collinearity problems well when combined with the SVM algorithm. Similar outcomes were also observed in the RR model. This may be because the RR algorithm cannot automatically select important feature variables when fitting sample data. Therefore, when the amount of input sample data is large, it is easily affected by outliers, thereby reducing the model’s predictive ability. It may also be that because determining optimal ridge parameters is critical when building an estimation model, poor parameter selection can lead to overfitting of the model, thereby reducing its interpretability (Smith and Campbell, 1980; Forrester and Kalivas, 2004). Moreover, this study demonstrates that the model achieves the highest estimation accuracy during the booting stage. This can be ascribed to the booting stage, which is the primary period for increases in rice chlorophyll and is characterized by high vegetation coverage and the absence of panicles. At this juncture, noise interference is minimized, and the spectral information collected is purer and more precise, providing advantageous conditions for estimating rice AGB (Chang et al., 2005; Takai et al., 2006; Kawamura et al., 2018).

## 5 Conclusion

This study implements SG smoothing and derivative transform for the preprocessing of spectral data, aiming to diminish fluctuations and noise interference. Subsequently, the Boruta and RFE algorithms are applied to select features from the first-order spectrum, thereby reducing the data dimensions and information redundancy and enhancing the data accuracy. A rice AGB estimation model was developed from the tillering stage to the maturity stage by integrating PLSR, PCR, SVM, and ridge regression machine learning algorithms. The key findings of the research are as follows:

(1) Except during the booting stage, FD exhibited the strongest correlation with AGB across the other four growth stages, followed by OS and then SD. As the rice growth stage progresses, the correlation shows a trend of first increasing and then decreasing.
(2) The performance of the Boruta algorithm is more stable, the selected characteristic bands are more sensitive, and effective dimensionality reduction of spectrum data is achieved. The number of feature strips provided by the RFE algorithm ranges from 3 to 439, and the number of feature strips selected by the Boruta algorithm is within 40.
(3) Among all model combinations, the characteristic band selected by the Boruta algorithm performs best, and FD-Boruta-PCR emerged as the superior model for estimating rice AGB, with R² values between 0.45 and 0.72 and RMSE values between 469.15 and 2392.65 kg/hm². The worst performing model is the FD-FB-SVM model, with R² values between 0.19 and 0.32 and RMSE values between 701.52 and 2980.00 kg/hm².
(4) As the progression from the tillering stage to the mature stage occurs, the accuracy of the AGB estimation model decreases. Among them, the booting stage was determined to be the most accurate prediction stage, as evidenced by an R² of 0.72 and an RMSE of 1290.24 kg/hm².

## Data availability statement

The original contributions presented in the study are included in the article/**Supplementary Material**. Further inquiries can be directed to the corresponding author.

## Author contributions

YN: Conceptualization, Writing – original draft, Writing – review & editing, Data curation, Formal Analysis. XS: Writing – review & editing. HY: Investigation, Writing – review & editing. YZ: Methodology, Supervision, Writing – review & editing. JL: Formal Analysis, Writing – review & editing. WW: Project administration, Writing – review & editing. YS: Validation, Writing – review & editing. QM: Visualization, Writing – review & editing. JKL: Resources, Writing – review & editing. XL: Funding acquisition, Writing – review & editing.
