# Supplementary material for: Estimation of the rice aboveground biomass based on the first derivative spectrum and Boruta algorithm
Source: Front Plant Sci. 2024 Apr 25;15:1396183. doi: 10.3389/fpls.2024.1396183 (PMC11079175; doi:10.3389/fpls.2024.1396183)
Supplement: Supplementary file 1 [file Table_1.docx]

Supplementary Material

**Appendix 1:** Rice AGB estimation models under different machine learning algorithms (MAE)

| **machine learning algorithm** | **Feature selection algorithm** | **Tillering** | | **Jointing** | | **Booting** | | **Heading** | | **Maturity** | |
| --- | --- | --- | --- | --- | --- | --- | --- | --- | --- | --- | --- |
|  |  | **Train Set** | **Test**  **Set** | **Train Set** | **Test**  **Set** | **Train Set** | **Test**  **Set** | **Train Set** | **Test**  **Set** | **Train Set** | **Test**  **Set** |
|  |  | **MAE**  **(kg/hm²)** | **MAE**  **(kg/hm²)** | **MAE**  **(kg/hm²)** | **MAE**  **(kg/hm²)** | **MAE**  **(kg/hm²)** | **MAE**  **(kg/hm²)** | **MAE**  **(kg/hm²)** | **MAE**  **(kg/hm²)** | **MAE**  **(kg/hm²)** | **MAE**  **(kg/hm²)** |
| PLSR | Boruta | 336.88 | 380.48 | 635.26 | 839.83 | 937.57 | 1142.62 | 918.11 | 1174.04 | 1579.91 | 1999.77 |
|  | RFE | 349.92 | 411.57 | 493.52 | 960.94 | 980.84 | 1145.73 | 734.94 | 1166.85 | 1358.82 | 1886.08 |
|  | Full band | 245.37 | 393.48 | 506.21 | 987.44 | 777.32 | 1228.16 | 771.22 | 1339.20 | 1363.31 | 1886.60 |
| PCR | Boruta | 335.91 | 384.28 | 669.07 | 841.66 | 941.82 | 1089.34 | 961.51 | 1098.01 | 1693.85 | 1937.47 |
|  | RFE | 351.04 | 412.46 | 860.46 | 1085.74 | 981.98 | 1148.04 | 989.39 | 1116.15 | 1704.87 | 2001.79 |
|  | Full band | 329.08 | 399.47 | 883.10 | 1115.07 | 982.75 | 1218.73 | 1148.51 | 1357.86 | 1733.17 | 2032.97 |
| SVM | Boruta | 277.80 | 357.02 | 483.53 | 812.17 | 606.66 | 1129.48 | 702.14 | 1209.68 | 762.21 | 1805.62 |
|  | RFE | 286.53 | 370.65 | 238.52 | 1114.98 | 839.55 | 1091.31 | 214.32 | 1316.73 | 494.29 | 2508.70 |
|  | Full band | 388.91 | 597.60 | 212.50 | 1161.43 | 383.37 | 1969.33 | 339.91 | 1971.74 | 591.40 | 2517.25 |
| RR | Boruta | 337.77 | 419.78 | 613.46 | 793.07 | 886.28 | 1106.12 | 805.59 | 1167.90 | 1443.99 | 1745.78 |
|  | RFE | 353.52 | 425.77 | 406.40 | 891.93 | 978.91 | 1101.08 | 494.68 | 1140.48 | 880.67 | 2041.70 |
|  | Full band | 157.54 | 426.73 | 414.49 | 917.34 | 534.13 | 1286.29 | 533.73 | 1400.88 | 882.40 | 2094.88 |
